# Optimal start in dialysis shows increased survival in patients with chronic kidney disease

**DOI:** 10.1371/journal.pone.0219037

**Published:** 2019-07-30

**Authors:** Araceli Caro Martínez, Antonio Olry de Labry Lima, José Manuel Muñoz Terol, Óscar Javier Mendoza García, César Remón Rodríguez, Leticia García Mochón, Pablo Castro de la Nuez, Nuria Aresté Fosalba

**Affiliations:** 1 Escuela Andaluza de Salud Pública (EASP), Campus Universitario de Cartuja, Granada, Spain; 2 CIBER en Epidemiología y Salud Pública (CIBERESP), Granada, Spain; 3 Instituto de Investigación Biosanitaria ibs, Granada, Hospitales Universitarios de Granada/Universidad de Granada, Granada, Spain; 4 Hospital Universitario Virgen del Rocío, Sevilla, Spain; 5 Hospital Universitario Puerto Real, Cádiz, Spain; 6 Coordinación Autonómica de Trasplantes de Andalucía (CATA), Sevilla, Spain; 7 Hospital Universitario Virgen Macarena, Sevilla, Spain; University of Mississippi Medical Center, UNITED STATES

## Abstract

**Objective:**

To compare the survival among patients with chronic kidney disease who had optimal starts of renal replacement therapy, dialysis or hemodialysis, with patients who had suboptimal starts.

**Methods:**

A retrospective cohort consisting of >18 year-old patients who started renal replacement therapy, using peritoneal dialysis or hemodialysis, in any public hospital or associated center of the Andalusian Public Health System, between the 1^st^ of January of 2006 and the 15^th^ of March of 2017. The optimal start was defined when all the following criteria were met: a planned dialysis start, a minimum of six-month follow-up by a nephrologist, and a first dialysis method coinciding with the one registered at 90 days. The information was obtained from the registry of the Information System of the Transplant Autonomic Coordination of Andalusia.

**Results:**

A total of 10,692 patients were studied. 4,377 (40.9%) of these patients died. A total of 4,937 patients (46.17%) achieved optimal starts of renal replacement therapy and showed higher survival rates (HR 0.669; 95% CI 0.628–0.712) in the multivariate analysis of Cox regression model.

**Conclusions:**

Patients with an optimal start of renal replacement therapy have a greater survival than those who had a non-optimal start. Therefore, the necessary measures should be encouraged to increase the optimal start of the patient in dialysis.

## Introduction

Chronic kidney disease (CKD) is a major public health concern associated with increased mortality and significant costs for the health system and for society. Thus, the cost associated with the care of this population amounts to 2.5% of the total health budget, although these patients represent <0.1% of the population [1,2]. This cost refers only to renal replacement therapy (RRT). The consumption of healthcare resources of patients with CKD on a global scale (approximately the 10% of the adult population) [3] is not well-known. In recent years there has been an increase in the prevalence of diabetes and the elderly population, which, along with other causes, is causing an increase in the number of patients with CKD [4,5]. In addition, it has been estimated that there is about 20% of the patients without diagnosis, and this proportion rises to 35–40% when it comes to patients with high prevalence diseases, such as hypertension or diabetes mellitus [6].

The literature describes that, in order to obtain the maximum benefit or the optimal benefit from the RRT, a timely referral to the nephrologist, timely preparation and initiation in dialysis is required [7]. A Cochrane review concludes that patients with CKD and late referrals to the nephrologist show a higher use of temporary catheters and emergency dialysis. On the contrary, these patients show a lower use of peritoneal dialysis and the anticipated transplant as a starting method. In addition, these patients experience higher rates of hospitalizations, more days of admission, and impose higher costs. On the other hand, it has been described that the type of vascular access in those treated with hemodialysis has an influence on the survival of the patient [8,9]. Taking into account the heavy burden of the disease, as a consequence of the high social and economic costs of RRT, priority attention is required to improve the care of these patients. In the light of this, the objective of this article is to compare the survival among patients who had optimal starts of RRT with patients who had suboptimal starts.

## Materials and methods

### Design

A retrospective cohort study.

### Study population

Patients included in the SICATA registry: adults (>18 years old) who started RRT in any hospital or associated center of the Andalusian Public Health System (APHS), in the modality of peritoneal dialysis (PD) or hemodialysis (HD), between the 1^st^ of January of 2006 and the 15^th^ of March of 2017 (date of data extraction).

### Exclusion criteria

Patients whose first option of treatment was the anticipated renal transplant (N = 339); patients treated by domiciliary HD (N = 20); patients who recovered renal function at some point of the monitoring (N = 251); patients treated at some point outside Andalusia and who subsequently returned to the APHS (N = 61); patients with indication of PD (ultrafiltration) for a refractory heart failure (N = 10); or patients who had more than two changes of dialysis therapy (N = 184).

### Variables to study

The dependent variable was the optimal start, which was defined by a consensus of experts as the simultaneous fulfillment of the following premises: definitive access to dialysis, planned dialysis start, a minimum of six-month follow-up by a nephrologist, and a first dialysis method coinciding with the one registered at 90 days. The failure to comply with any of these criteria was classified as suboptimal start. In addition, the following independent variables were considered:

1. Sociodemographic variables: sex, age at treatment initiation, employment status, place born and province of start of RRT.
2. Clinical variables: date of treatment start, Charlson index at the beginning of RRT, primary renal disease and exitus.

### Process

The information was extracted from the Basic Module of the Chronic Renal Insufficiency (CRI) Subsystem of the Information System of the Transplant Autonomic Coordination of Andalusia (SICATA) [5], which is a public and compulsory registry of patients in RRT in Andalusia. Inclusion in this registry requires duly signed informed consent. The extracted information did not include identifiable data of the patient, following the European Union regulations on personal data protection. All professionals participating in the study signed a commitment of data confidentiality and an ethical commitment.

### Measurement instruments

The Charlson Comorbidity Index began to be registered in the CRI module from the year 2006 and it is collected at the beginning of the first RRT. Therefrom, 19 medical situations that are pondered between 1 and 6 can be obtained.

### Statistical analysis

A descriptive analysis of the variables was carried out with the most commonly used statistics: proportions and percentages, for the categorical variables, and averages and standard deviations for the continuous variables. First, an analysis was performed to describe the differences between optimal and suboptimal starts. For this purpose, the qualitative variables were contrasted using the chi-squared test, and the quantitative variables using the logistic regression model. Subsequently, a bivariate survival analysis was performed through the Kaplan-Meier method and using the log-rank test for comparison. The survival time was defined as from the start in RRT until the death of the patient or censoring. The proportional hazards assumption was verified using the Schoenfeld Test. The statistical significance was defined as error probability α (5%) and 95% confidence intervals (95% CI). Finally, a Cox regression model was constructed with the independent variables that were shown statistically different survival. All statistical analyses were performed with Stata v13.

## Results

**Fig 1:**
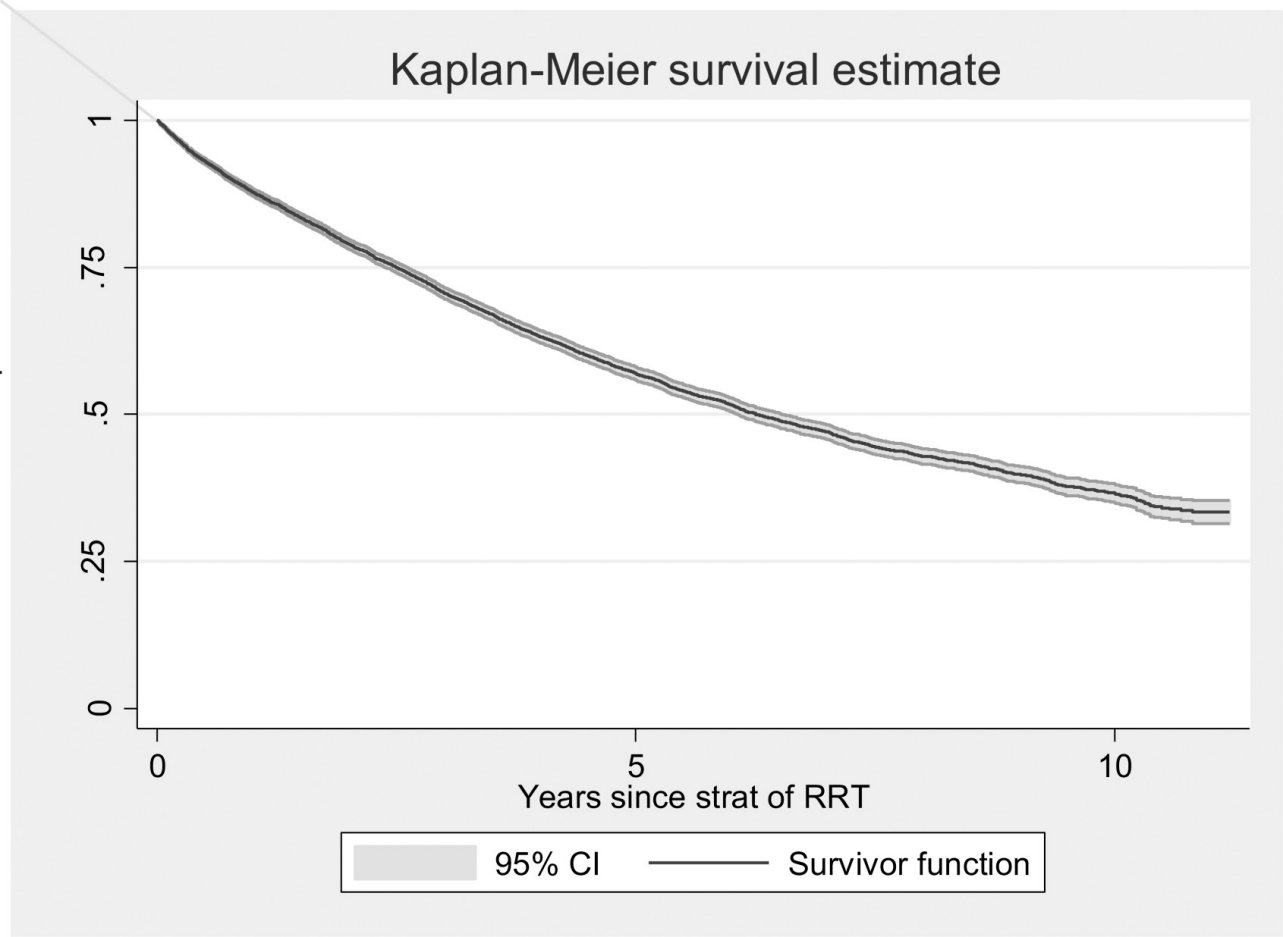
Survival function of the total number of patients included in the study.

**Fig 2:**
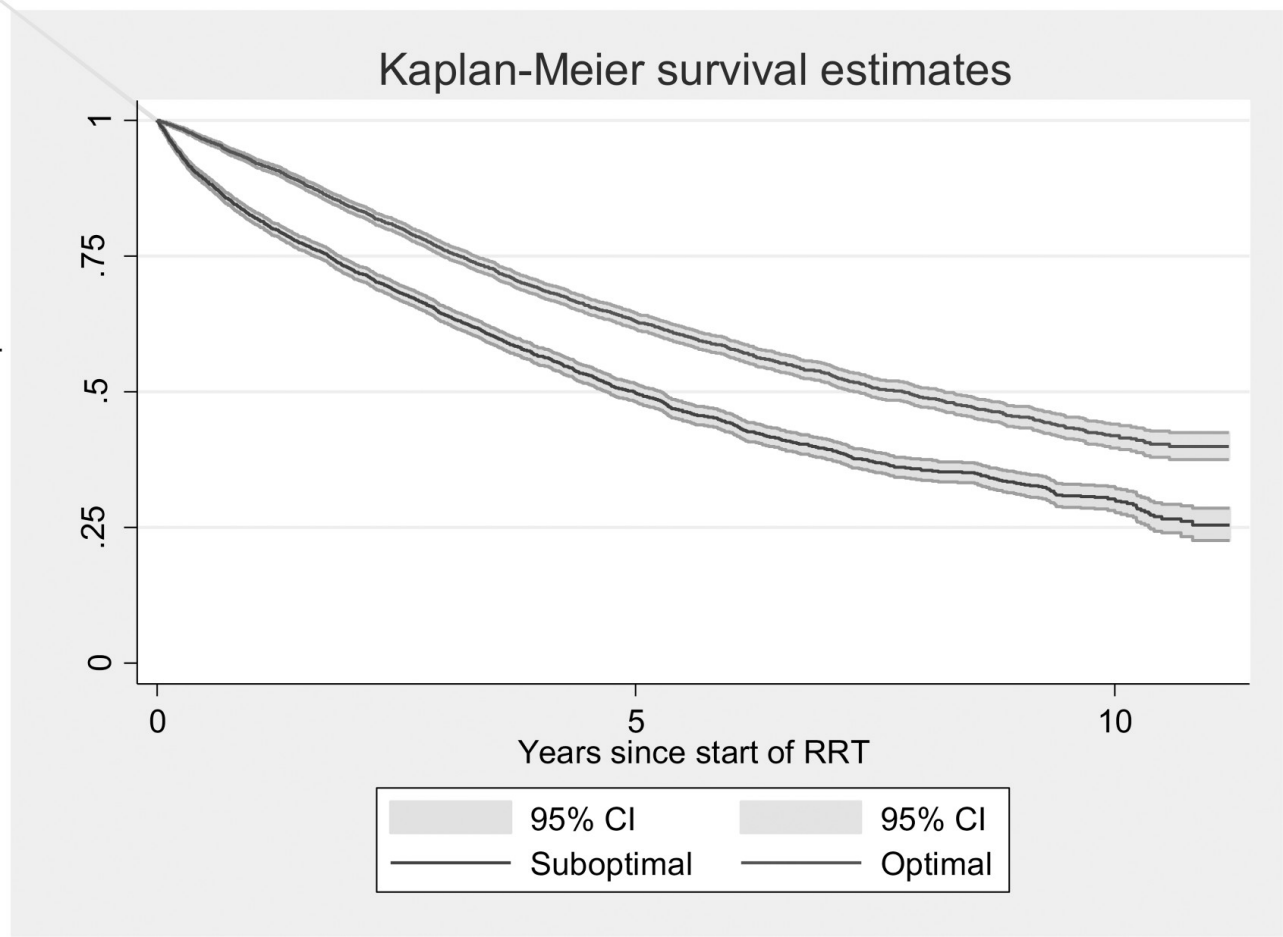
Survival function according to the optimal start in RRT.

After applying the exclusion criteria, the cohort was composed of 10,692 patients, of which 4,937 (46.17%) patients achieved optimal starts and 5,755 (53.83%) achieved suboptimal starts. A total of 4,377 (40.9%) of the patients died during the monitoring, with a median survival time of 6.25 years (95% CI 6.06–6.51). Fig 1 shows the survival function of the total number of patients included in this study. Fig 2 shows the survival curve according to the optimal start. The median survival for patients with optimal start was 7.79 years (95% CI 7.29–8.22) and 4.98 years (95% CI 4.70–5.25) for patients with suboptimal start.

At a bivariate level, statistically significant differences were observed between patients who achieved optimal and suboptimal starts in the variables of employment status (p<0.001), place born (p<0.001), diabetes (p<0.001), primary renal disease (p<0.001), exitus (p<0.001) and Charlson Comorbidity Index (p<0.001) (Table 1).

**Table 1:** Patient socio-demographic and clinical characteristics according to dialysis optimal initiation and (n = 10,692), and survival of those variables.

Variable		Suboptimal N (%)	Optimal N (%)	p†	HR (95%CI)^¥^
Sex	Male	3,125 (63.3%)	3.547 (61.63%)		
	Female	1,812 (36.70%)	2,208 (38.37%)	0.077	0.914 (0.859–0.972)
Employment status	Not active	2,530 (51.25%)	2,997 (52.08%)		
	Active	512 (10.37%)	855 (14.86%)		0.333 (0.293–0.378)
	Lost	1,895 (38.38%)	1,903 (33.07%)	<0.001	0.869 (0.811–0.932)
Place born	Spain	4,327 (87.84%)	5,239 (91.11%)		
	Rest of Europe	121 (2.46%)	93 (1.62%)		1.173 (0.945–1.457)
	Africa	139 (2.82%)	69 (1.20%)		0.374 (0.269–0.519)
	America	78 (1.58%)	55 (0.96%)		0.370 (0.243–0.563)
	Asia/Oceania/ Lost	261 (5.30%)	294 (5.11%)	<0.001	1.092 (0.967–1.235)
Diabetes	Yes	2,014 (40.79%)	1.962 (34.09%)		
	No	2,923 (59.21%)	3,793 (65.91%)	<0.001	0.639 (0.602–0.679)
Primary renal disease	Glomerular Disease	556 (11.26%)	591 (10.28%)		
	Tubulointerstitial Dis.	445 (9.01%)	607 (10.56%)		1.554 (0.331–1.815)
	Systemic Disease	2,243 (45.43%)	2,380 (41.39%)		2.032 (1.787–2.310)
	Familial Nephropathies	201 (4.07%)	668 (11.62%)		0.810 (0.667–0.983)
	Other	1,492 (30.22%)	1,504 (26.16%)	<0.001	1.656 (1.449–1.893)
Exitus	Live	2,610 (52.87%)	3,705 (64.38%)		
	Death	2,327 (47.13%)	2,050 (35.62%)	<0.001	NA
Variable		Suboptimal Mean (sd)	Optimal Mean (sd)	p	HR (95%CI)
Age at treatment initiation (years)		63.07	63.16	0.208*	1.040 (1.037–1.043)
Charlson Comorbidity Index		5.95	5.36	<0.001*	1.223 (1.209–1.237)

*Mann-Whitney test

† chi2 differences between optimal and suboptimal start

ACKD: advanced chronic kidney disease; HD: hemodialysis; PD: peritoneal dialysis

¥ HR (95%CI): hazard ratio (95% confidence interval). At the bivariate level, HR indicates survival according to the different sociodemographic and clinical characteristics.

sd: standard deviation

In terms of survival, at a bivariate level, a better survival was found associated with being a woman, underage, with active employment status, lower score in the Charlson Index and not having a diabetes diagnosis (p<0.001) (Table 1).

Patients with optimal start of RRT showed greater survival than those with suboptimal start (HR 0.635; 95% CI 0.598–0.674). Thus, in the multivariate model, when adjusting by the rest of the variables, this risk was (HR 0.669; 95% CI 0.628–0.712). Another variable significantly associated with greater survival in the multivariate model was having an active employment status (HR 0.626; 95% CI 0.545–0.719). On the contrary, the variables associated with worse survival rates were having diabetes (HR 1.191; 95% CI 1.100–1.289), an older age (HR 1.030; 95% CI 1.026–1.034), higher Charlson Index scores (HR 1.178; 95% CI 1.157–1.200) at treatment initiation, and having Tubulointerstitial Disease (HR 1.320; 95% CI 1.128–1.544), Systemic Disease (HR 1.448; 95% CI 1.266–1.655) or others (HR 1.206; 95% CI 1.053–1.382) as primary renal disease (Table 2).

**Table 2:** Crude and adjusted hazard ratios of Cox regression models of the optimal start (n = 10,671).

Crude Model		p	HR (95%CI)*
Optimal Start	Suboptimal		
	Optimal	<0.001	0.635 (0.598–0.674)
Adjusted Model*		p	HR (95%CI)*
Optimal Start	Suboptimal		
	Optimal	<0.001	0.669 (0.628–0.712)
Sex	Male		
	Female	0.331	0.969 (0.910–1.032)
Start Method	HD		
	PD	0.244	0.934 (0.834–1.047)
Place born	Spain		
	Rest of Europe	0.015	1.311 (1.053–1.634)
	Africa	0.032	0.695 (0.499–0.968)
	America	0.029	0.625 (0.410–0.953)
	Asia/Oceania/ Lost	<0.001	1.297 (1.138–1.478)
Province of start of the RRT	P1		
	P2	0.637	1.025 (0.924–1.136)
	P3	0.109	1.087 (0.981–1.205)
	P4	0.110	1.102 (0.978–1.242)
	P5	0.001	1.233 (1.092–1.391)
	P6	<0.001	1.374 (1.206–1.565)
	P7	0.313	1.070 (0.937–1.221)
	P8	<0.001	1.319 (1.149–1.515)
	No data	0.009	1.221 (1.050–1.420)
Employment status	Not active		
	Active	<0.001	0.626 (0.545–0.719)
	Lost	0.012	0.901 (0.813–0.977)
Primary renal disease	Glomerular Disease		
	Tubulointerstitial Disease	0.001	1.320 (1.128–1.544)
	Systemic Disease	<0.001	1.448 (1.266–1.655)
	Familial Nephropathies	0.095	0.846 (0.696–1.029)
	Other	0.007	1.206 (1.053–1.382)
Diabetes	No		
	Yes	<0.001	1.191 (1.100–1.289)
Age at treatment initiation (years)		<0.001	1.030 (1.026–1.034)
Charlson Comorbidity Index		<0.001	1.178 (1.157–1.200)

ACKD: advanced chronic kidney disease; HD: hemodialysis; PD: peritoneal dialysis

* HR (95%CI): hazard ratio (95% confidence interval)

## Discussion

This study compares the survival of patients who started RRT with a planned peritoneal dialysis or hemodialysis, with a definitive dialysis access, a minimum of six-month follow-up by a nephrologist, and a first dialysis method coinciding with the one registered at 90 days (all indicators of optimal start), with patients who did not comply with the criteria (suboptimal start). Thus, the analyses performed show that patients who achieved an optimal start in dialysis have, on average, a survival rate of 2.8 years higher than patients who had a suboptimal start. This difference has been shown to be statistically significant.

The proportion of patients who achieved an optimal start of RRT is within the range of values described in the literature [10–17]. In this sense, the literature provides heterogeneous considerations of what is the optimal start of the patient in dialysis means. However, most definitions associate the concept with a planned start. This paper provides a wider (though at the same time restrictive) definition of what it is an optimal start, as a result of the consensus reached by the working team which includes nephrologists. This definition integrates other requirements such as the coincidence in the registry of the treatment at 90 days. It would have been interesting to incorporate as a requirement the training and information of the patient on the available options for RRT [18–20]. Unfortunately, the database did not enable the identification or assessment of the training received by the patient. This training improves patients’ adherence to recommendations and healthy habits. Thus, a systematic review of the educational interventions for patients with CKD states that the educational interventions can improve the results of the patient, both those informed by them (quality of life) and others of a clinical nature (estimated glomerular filtration rate, survival, etc.) [21]. In addition, it must be taken into consideration that patients at the beginning of RRT feel an important socioemotional and psychological burden, so this training offers them tools to obtain answers to this need [22], as well as helping them in the decision-making process and comprehension of the treatment options.

Patients who have a suboptimal start of RRT have a lower probability of being included in the waiting list for a renal transplant and a lower probability of starting treatment with PD [23]. In addition, it must be taken into consideration that patients who started PD show a higher probability of receiving renal transplantation. It is important to bear in mind that the benefits of receiving renal transplantation, in terms of survival, are greater, and this should be taken into consideration in the interpretation of the results.

On the other hand, cases in which patients, in the course of their disease, start dialysis late, or have an unexpected renal deterioration leading to an urgent start, are examples of suboptimal starts and have negative consequences on their well-being and quality of life. This hampers the adequate selection of patients for the RRT since a “shared decision making” is not possible and can significantly influence the percentage of use of the different dialysis modalities and the probability for renal transplantation. In addition, these conditions make anticipated renal transplant impossible and hinders the inclusion in the waiting list at an appropriate time.

According to the health technology assessment report carried out in Spain, the cost of the planned-start is approximately € 3,300 cheaper than the urgent-start. In the literature these figures increase up to $ 13,200 [24].

In light of the above, it would be desirable and necessary to increase the resources allocated to primary care of patients with CKD. This will be key to implement educational interventions (training programs in risk assessment and management) to delay the progression of the disease and reduce late referrals to nephrologists, among others. In hospitals settings, there is a need for specialized consultations provided by a multidisciplinary team (at least a nephrologist, specialized nursing and structured programs for patient education and training), for patients with advanced chronic kidney disease (ACKD) [25,26]. These and other measures are key to promote an increase in the percentage of patients achieving an optimal start in dialysis, according to the results of this work and taking into consideration the high clinical and economic impact on patients and on the health system.
